# The Role of Glycosphingolipids in Immune Cell Functions

**DOI:** 10.3389/fimmu.2019.00090

**Published:** 2019-01-29

**Authors:** Tao Zhang, Antonius A. de Waard, Manfred Wuhrer, Robbert M. Spaapen

**Affiliations:** ^1^Center for Proteomics and Metabolomics, Leiden University Medical Center, Leiden, Netherlands; ^2^Department of Immunopathology, Sanquin Research, Amsterdam, Netherlands; ^3^Landsteiner Laboratory, Amsterdam UMC, University of Amsterdam, Amsterdam, Netherlands

**Keywords:** glycans, glycolipids, regulation, expression, immunity, differentiation, receptors, cancer

## Abstract

Glycosphingolipids (GSLs) exhibit a variety of functions in cellular differentiation and interaction. Also, they are known to play a role as receptors in pathogen invasion. A less well-explored feature is the role of GSLs in immune cell function which is the subject of this review article. Here we summarize knowledge on GSL expression patterns in different immune cells. We review the changes in GSL expression during immune cell development and differentiation, maturation, and activation. Furthermore, we review how immune cell GSLs impact membrane organization, molecular signaling, and trans-interactions in cellular cross-talk. Another aspect covered is the role of GSLs as targets of antibody-based immunity in cancer. We expect that recent advances in analytical and genome editing technologies will help in the coming years to further our knowledge on the role of GSLs as modulators of immune cell function.

## Introduction

The surface of cells is covered with glycans (or carbohydrates) that are part of glycoproteins, glycosaminoglycans, or glycosphingolipids (GSLs). GSLs consist of glycans conjugated to a lipid (ceramide) core and comprise a diverse group of over 300 different complex molecules based on variation in the glycan buildup (1–3). The diversity of glycan structures on GSLs is directed by a range of proteins involved in glycan biosynthesis including glycosyltransferases (GTs), glycosidases, enzymes involved in glycan precursor biosynthesis and nucleotide sugar transporters. These proteins are differentially expressed throughout the immune system giving rise to a large variability in GSL expression patterns which suggests a functional role for GSLs in immune cell development or activation (4). GSLs are essential parts of GSL enriched microdomains (GEMs) in the cell membrane, which have an important role in molecular signaling, cellular cross-talk, and cell adhesion (5–7). Consequently, mice deficient in subclasses of GSLs show immunological, reproductive, neuronal, renal, gastrointestinal, and metabolic defects (8). To date, cell surface GSLs have been shown to be involved in diverse immune processes, including differentiation, immune recognition, and transduction of activation signals. In this review, we summarize the literature on GSL expression of various immune cells and highlight the functions that have been attributed to these GSLs.

## Biosynthesis and Expression of GSLs in Naïve and Differentiated Immune Cells

**Figure 1:**
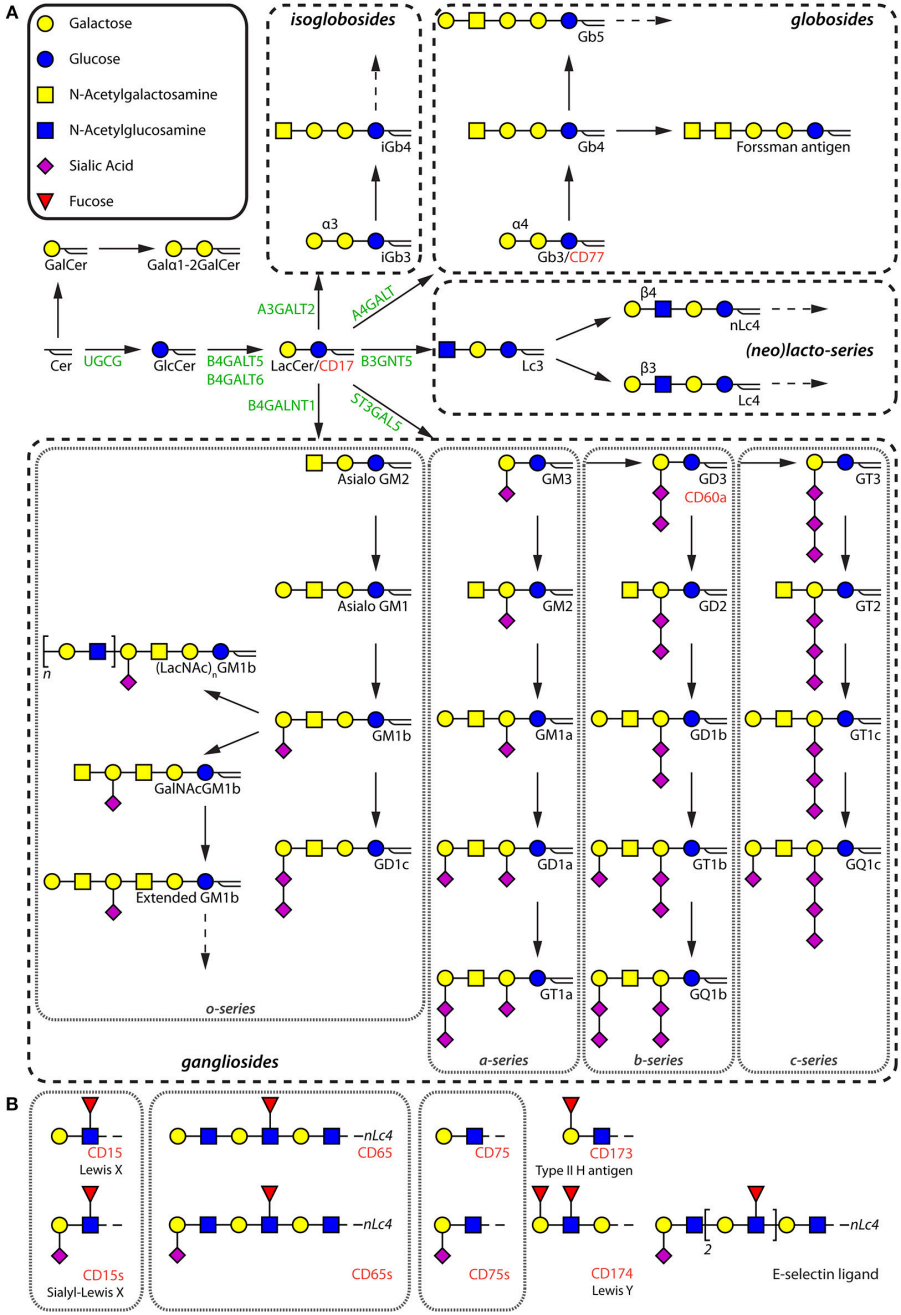
Schematic diagram of the different types of GSLs. **(A)** Major GSLs expressed in immune cells and proposed GSL biosynthetic pathway. The key enzymes are in green. GSLs that have been given a cluster of differentiation (CD) number are annotated in red. **(B)** Terminal glycan motifs that have been given a CD number and the most prominent E-selectin ligand present on human neutrophils.

GSLs are divided into two groups based on the presence of a galactosylated or glucosylated ceramide (Cer) core. The latter group consists of complex structures subdivided into gangliosides, (iso)globosides, and (neo)lacto-series GSLs (Figure 1A; Table S1). The GTs UDP-glucose ceramide glucosyltransferase (UGCG) and β1,4-galactosyltransferase 5/6 (B4GALT5/6) synthesize glucosylceramide (GlcCer) and lactosylceramide (LacCer) respectively, forming the precursor of GlcCer-based GSLs (8). GSLs are further divided into four major series based on the synthesis pathways (Figure 1A). Alpha2,3-sialyltransferase 5 (ST3GAL5) is the key enzyme for the synthesis of GM3, which is the parent structure for *a*-, *b*-, and *c*-series gangliosides. β1,4-*N*-acetylgalactosaminyltransferase 1 (B4GALNT1) catalyzes the generation of asialo GM2 by transferring *N*-acetylgalactosamine (GalNAc) to LacCer, initializing the synthesis of *o*-series gangliosides. Lactotriaosylceramide (Lc3) is the starting structure for synthesis of (neo)lacto-series GSLs, which is synthesized by β1,3-*N*-acetylglucosaminyltransferase 5 (B3GNT5). The (iso)globosides globotriaosylceramide (Gb3) and isoglobotriaosylceramide (isoGb3) are generated by the addition of a galactose to LacCer in α1,4 and α1,3 linkages by α1,4-galactosyltransferase (A4GALT) and α1,3-galactosyltransferase 2 (A3GALT2) respectively (Figure 1A). Further extension and modifications of these core structures, including elongation, sulfation, and sialic acid acetylation, contributes to the diversity of the repertoire expressed in (immune) cells (9–13).

**Figure 2:**
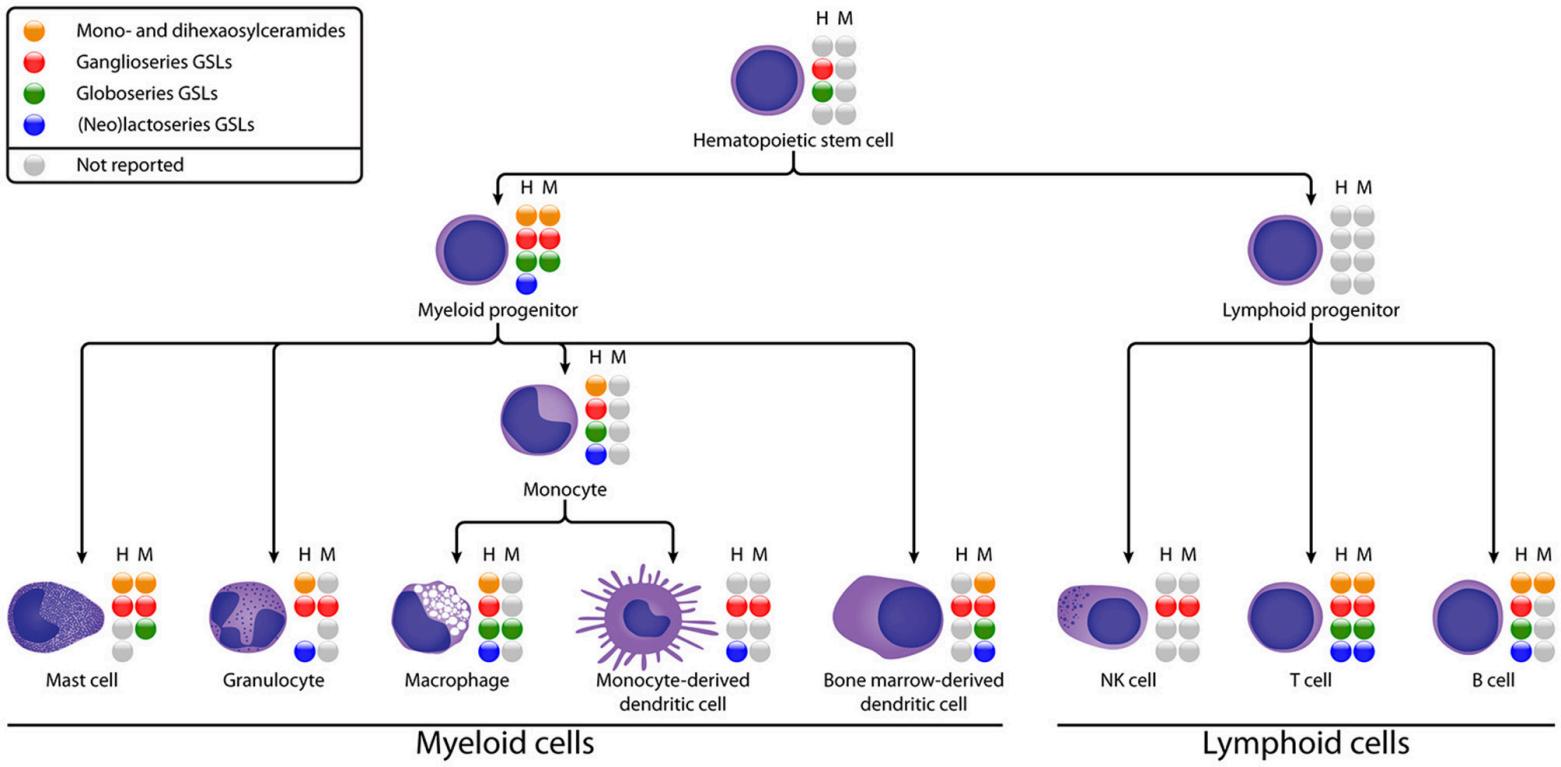
Schematic diagram of GSL expression in different stages of human (H) and murine (M) immune cell differentiation. GSL subsets that have been reported are represented by colored spheres, unreported subsets are represented by gray spheres. The absence of a sphere indicates that the GSL subset could not be detected. See Table 1 for details on the subset expression.

The GSL repertoire of different immune cells varies per cell type under physiological conditions (14–16). The expression of some GSLs on immune subsets is well-studied, and antibodies against them have found their way into the cluster of differentiation (CD) marker set established decades ago. At that time, it was not yet known that these antibodies recognized glycan headgroups of GSLs, and therefore they have been assigned a specific CD-number. This group includes CD15, CD17, CD60, CD65, CD75, CD77, CD173, and CD174 (Figures 1A,B), some of which are still being used to phenotype and isolate immune cell subpopulations (17). For example, CD77 represents the Gb3 structure, which has been employed to define a B cell subpopulation. Notably, the specific terminal glycan motifs of CD15, CD75, CD173, and CD174, can be carried by GSLs and glycoproteins. In the following sections, we summarize current knowledge on GSL expression patterns in different immune cell subsets (see Figure 2 and Table 1 for an overview).

**Table 1:** GSL expression in human and murine immune cells.

		**GSL types**				
**Cell type**	**Sources**	**GlcCer, LacCer, GalCer**	**Ganglioside**	**Globosides**	**(neo)Lacto-series**	**References**
HSCs	Human	N.R.	GM3	Gb5	N.R.	(18, 19)
	Mouse	N.R.	N.R.	N.R.	N.R.	N.R.
Myeloid progenitors	Human	GlcCer, LacCer	GM3^a^, GM2, GD3	Gb3, Gb4	Lc3^b^, (n)Lc4^b^	(20–23)
	Mouse	LacCer	GM1^a^, GD1a^a^, GM2, GD3, GM3, GD1b	Gb3^b^	N.D.	(24)
Mast cells	Human	N.R.	GD3^a^, GM3	N.R.	N.R.	(25, 26)
	Mouse	GlcCer, LacCer	GM1^a^, GM3^a^, asialo GM1	Gb3, Gb4, Fo	N.D.	(27–30)
Maturated mast cells	Human	LacCer	GD3^a^, GM3, and *a*-series ganglioside (GM2, GM1, GD1a)^c^	N.R.	N.R.	(25)
	Mouse	GlcCer	GM3^a^	N.R.	N.D.	(29, 30)
Neutrophils	Human	GlcCer,LacCer^d^, GalCer	GM1^a^, GM3^a^, complex type, (no GD3)	N.D.	Lc3^d^, nLc4, nLc6, S(3)nLc4, S(6)nLc4, S(3)nLc6	(31–40)
	Mouse	N.R.	N.R.	N.R.	N.R.	N.R.
Eosinophils	Human	N.R.	GM1	N.R.	N.R.	(41, 42)
	Mouse	N.R.	N.R.	N.R.	N.R.	N.R.
Basophils	Human	N.R.	N.R.	N.R.	N.R.	N.R.
	Mouse	N.R.	Asialo GM1	N.R.	N.R.	(43)
Monocytes	Human	GlcCer, LacCer	GM3^a^	(iso)Gb3^d^, Gb4^d^	Lc3^b^,(n)Lc4^b^, S(3)nLc4, S(6)nLc4, S(3)nLc6	(36, 44–48)
	Mouse	N.R.	N.R.	N.R.	N.R.	N.R.
Macrophages	Human	GlcCer, LacCer	GM3^a^	Gb3^d^, Gb4^d^, Gb5	Lc3^b^, (n)Lc4^b^, S(3)nLc4, S(6)nLc4, S(3)nLc6	(44, 45, 48–52)
	Mouse	N.R.	N.R.	Gb3^d^, Gb4^d^, Gb5, Fo^c^	N.R.	(53, 54)
moDCs	Human	N.R.	GM3^a^	N.R.	Lc3, nLc4	(55, 56)
	Mouse	N.R.	GM3^a^	N.R.	N.R	(56)
BMDCs	Human	N.R.	GM3^a^	N.R.	N.R.	(56)
	Mouse	LacCer, Galα1-2, GalCer	GM3^a^, complex type, (*a-, b-* and *o-*series),asialo GM1, asialo GM2	(iso)Gb3, (iso)Gb4, Gb5, Fo	Lc3	(57)
B cells	Human	GalCer, GlcCer, LacCer	GM3^a^, complex type (*a-, b-* and *o-*series), asialo GM1, asialo GM2, GD3, 7-*O*-GD3 and 9-*O*-GD3	Gb3^d^, Gb4^d^	Lc3^b^, nLc4^b^	(12, 58–64)
	Mouse	GalCer, GlcCer, LacCer	N.R.	N.R.	N.R.	(65)
T cells	Human	GlcCer, LacCer	GM1^a^, GM3^a^, complex type (*a-, b-* and *o-*series), GD3, 7-*O*-GD3, 7-*O*-GD3	Gb3^d^, Gb4	nLc4	(13, 18, 58, 61, 66, 67)
	Mouse	GlcCer, LacCer	GM1^a^, GM3^a^, complex type (*a-, b-* and *o-*series), asialo GM1, extended GM1b (more complex than human)	(iso)Gb3, (iso)Gb4	Lc3	(57, 66, 68–71)
NK cells	Human	N.R.	Asialo GM1, 7-*O*-GD3	N.R.	N.R.	(50, 72)
	Mouse	N.R.	Asialo GM1, GM1	N.R.	N.R.	(69, 73, 74)

N.R., Not reported; N.D., Not detected;

a Dominant abundance;

b Low abundance;

c Specific expression;

d *Dominant abundance among neutral GSLs*.

### Hematopoietic Stem and Progenitor Cells

HSCs are multipotent cells located in bone marrow which can differentiate into myeloid and lymphoid progenitor cells (Figure 2). To date, the GSL content of HSCs has hardly been studied, probably due to the low abundance of HSCs in blood and bone marrow and the difficulty to isolate them (75). Some studies suggest the presence of GM1 on HSCs based on binding of Cholera Toxin B (CTB). However, this glycan-binding subunit B of cholera toxin has a broader specificity then just GM1 (discussed in section Organization of Membrane Microdomains) (76–78). Furthermore, Giebel et al. found that GM3 is expressed and localized at the leading edge of polarized CD34^+^ HSCs, suggesting a role for GM3 GEMs in the polarization of HSCs (18). With respect to neutral GSLs, Gb5 was detected after exposure to fetal calf serum (19), but not on freshly isolated HSCs, even not as a sialylated or fucosylated variant. This finding is supported by a lack of expression of the relevant GTs in HSCs. Thus, environmental factors may change the expression of GTs, which has to be kept in mind when evaluating GSL expression on cultured or stimulated cells. In addition, CD173 and CD174 (Figure 1B), which may be carried by GSLs, are found to be specifically expressed on naïve CD34^+^ HSCs and disappear after differentiation (79).

In human myeloid progenitors—which give rise to mast cells, granulocytes, monocytes, and bone marrow-derived dendritic cells—GlcCer, LacCer, gangliosides (GM2, GM3, and GD3), and globosides (Gb3 and Gb4) are reported (Figure 2). In some studies, (neo)lacto-series GSLs (Lc3 and nLc4) were also weakly detected (20, 23). The mouse myeloid progenitor cell line FDC-P1 displays LacCer, gangliosides (GM1, GM2, GM3, GD1a, GD1b, and GD3), and globoside Gb3, while no GlcCer or (neo)lacto-series GSLs were detected (24). This work further revealed that GM1 and GD1a are the two major gangliosides accumulated by FDC-P1 cells. Reports on GSL expression of lymphoid progenitors, the precursors of NK, T and B cells, are absent in literature. We can conclude that gangliosides are expressed in HSCs and progenitor cells, while globosides and (neo)lacto-series GSLs are hardly expressed in HSCs, and at relatively low levels during further differentiation.

### Myeloid Cells

Myeloid cells have been studied for decades and express some unique GSLs. The best described myeloid-specific GSL is a fucosylated neolacto-series GSL which is known as the CD65 antigen (Figure 1B) (80–82). It is expressed on most myeloid cells during development, highly on granulocytes and weakly on monocytes in peripheral blood. The sialylated form of CD65 (CD65s) is expressed when the myeloid progenitor antigen CD34 disappears, indicating that CD65s expression marks a turning point in myeloid cell differentiation. In addition to CD65 and CD65s, the expression patterns of other GSLs in mast cells, granulocytes, monocytes, macrophages, and DCs will be discussed in the following sections (Figure 2).

#### Mast Cells

After development from bone marrow-derived progenitor cells, mast cells can circulate as CD34^+^ progenitor cells, or migrate into tissues to differentiate into mature mast cells under the influence of cytokines.

It is well-recognized that GD3 is the most abundantly expressed GSL on the surface of nearly all mast cells (26). Zuberbier et al. studied the alterations of ganglioside expression during maturation of the human mast cell line HMC-1. Upon differentiation, a highly elevated expression of GM3 and GM3-derived *a*-series gangliosides (Figure 1A), including GM2, GM1a, and GD1a, were observed as a result of upregulation of the GTs ST3GAL5, B4GALNT1, ST8SIA1, and ST3GAL2 (25). Similarly, mouse serosal mast cells (SMCs) mainly express GM3. The ability to synthesize complex acidic GSLs is possibly lost during mast cell maturation, because *in vitro* differentiated bone marrow-derived mast cells (BMMCs) expressed—next to GM3—GM1, which was lost when matured toward SMC-like cells (29, 30).

Neutral GSLs have not been biochemically analyzed in human mast cells, except for the observation of LacCer in HMC-1 cells (25). For the murine BMMCs, expression of GlcCer, LacCer, asialo GM1, Gb3, and Gb4 has been described, while no (neo)lacto-series GSLs have been reported (27, 28, 83, 84). Interestingly, specifically Gb4 was found to be expressed in secretory granules, where it may have a yet unknown function (28). During *in vitro* activation of BMMCs, surface expression levels of Gb4 increased, which is thought to be the result of the fusion of internal membranes with the plasma membrane (28). Intriguingly, the Forssman glycolipid antigen (Fo), GalNAcα1-3Gb4, is specifically expressed by SMCs and not by BMMCs (27). In contrast to murine cells, only Gb5, but not LacCer, Gb3 or Gb4, was found on rat SMCs (85).

#### Granulocytes

Neutrophils, eosinophils, and basophils are granulocytes derived from myeloid precursor cells and have similar characteristics and functions in innate immune responses.

Human neutrophils are rich in GSLs, and around 2 mg of GSLs can be extracted from 10^10^ cells. Detailed structural characterization of these GSLs showed neutrophils contain a very complex ganglioside mixture (34, 37, 86, 87). Similar to BMMCs, GM1 and GM3 are the most abundant gangliosides in neutrophils. Compared to other bone marrow-derived cells, mature neutrophils were found to express the highest levels of GM1 (32, 35, 87). Later studies revealed that the presence of GM1 is related to the stage of neutrophil apoptosis, allowing the use of GM1 as an aging marker for neutrophils (40). In contrast to mast cells, neutrophils were not found to express GD3 (34).

With respect to neutral GSLs, human neutrophils express GlcCer, LacCer, and a set of (neo)lacto-series GSLs, but no globoside has been detected (23, 31–33, 35, 39, 88). During differentiation of the promyelocyte cell line HL60 toward granulocytes using all-trans retinoic acid or phorbol myristate acetate (PMA), the (neo)lacto-series synthase B3GNT5 was upregulated (21, 89). Therefore, Lc3, after LacCer, appeared to be the predominant species accounting for about 10% of the total neutral GSL fraction (38, 90). Notably, the neolacto-series GSLs are the major class in neutrophils, containing Lc3, nLc4, nLc6, and *a*-series of GSLs carrying Le^x^ (Lewis X structures, Galβ1-4(Fucα1-3)GlcNAcβ1-), also known as CD15 (Figure 1B) (35, 38). In addition, sialylated neolacto-series GSLs (S(3)nLc4, S(6)nLc4, and S(3)nLc6) have also been detected (33, 91). The unique expression of these neolacto-series GSLs by neutrophils in comparison to other immune cells may be required to interact with pathogens or the humoral immune system.

To date, there are hardly any studies on the GSL expression of eosinophil and basophils. Ganglioside GM1 has been detected at the surface of eosinophils, and a stepwise upregulated expression was observed during cell differentiation from the promyelocyte to the eosinophil stage (41, 42). For murine basophils, a high level of asialo GM1 expression has been described (43).

#### Monocytes, Macrophages, and Dendritic Cells

Monocytes, macrophages, and dendritic cells (DCs) are phagocytic innate immune cells, which drive adaptive immune responses via antigen processing and presentation (92, 93). Monocytes can differentiate *in vitro* into macrophages or monocyte-derived DCs (moDCs) after specific cytokine stimulation. All monocytes, macrophages, and moDCs express high levels of GM3 in both human and mouse (49, 94, 95). Cultured human macrophages yield approximately seven times more GM3 per million cells than *ex vivo* peripheral blood monocytes (2.7 vs. 0.4 μg respectively) (46). Accordingly, such macrophages, but also *in vitro* differentiated moDC express 10-fold higher ST3GAL5 levels compared to freshly isolated monocytes (46, 55, 56, 96). Interestingly, the high expression of acidic GSLs is probably in part also facilitated by a decreased expression of α2,3- and α2,6-sialidases (such as NEU3), which was for example observed in PMA-differentiated THP-1 macrophages (97, 98). Similar to humans and mice, rat abdominal macrophages express GM3 as the predominant acidic GSLs, followed by GM2 (85).

Monocytes and macrophages seem to have a different neutral GSL composition compared to other human myeloid immune cells since they express globosides ((iso)Gb3 and Gb4) as the major neutral GSLs (36, 44, 45, 48, 52). Neolacto-series GSLs such as Lc3 and nLc4 are also detectable and upregulated during differentiation toward moDCs, but are reduced during differentiation toward macrophages as a result of decreased B3GNT5 gene expression (36, 44, 45, 55, 96). Additionally, during macrophage differentiation the expression of Gb5 is upregulated, which—like Gb3—is a target for the human immunodeficiency virus (HIV) gp120 glycoprotein (94, 99). In mouse abdominal macrophages, it has been demonstrated that neutral GSLs are expressed at higher levels than gangliosides. Asialo GM1 was specifically expressed after a 3-day culture, but its expression gradually declined after prolonged cultures. Other neutral GSLs including GlcCer and Gb3 were highly upregulated in macrophage differentiated murine M1 cells (100–102). Fo GSLs are expressed in mature mouse macrophages and increases during the lifetime of the cell. It is used as a differentiation marker and is specifically expressed in defined areas in spleen, lymph nodes, and bone marrow, which suggests it may have a function in lymphoid organ homing or residency (53, 54, 103–105). In addition to the globosides Gb3, Gb4, and Gb5, the specific neutral GSL Galα1-3(F(2))ASGM1 was also found to be highly expressed in rat macrophages (85).

During differentiation of murine bone marrow precursors to bone marrow-derived DCs (BMDCs), no significant change in acidic GSLs nor LacCer or asialo GM1 content was found, even though *a*-series (GM1a, GD1a, and GT1a), *b*-series (GD3, GD1b, and GT1b), and *o*-series (asialo GM1 and GM1b) are generally present in BMDCs (57). However, Lc3, Gb3, Gb4, and Fo GSLs were found to be more abundant on mature BMDCs. Interestingly, Li et al., also described the presence of isoGb3 and isoGb4 to be enhanced in mature BMDC. Though the isoGb3 expression level was very low compared to Gb3, ~0.8% in both immature and mature DCs. IsoGb3 can be specifically recognized in the context of CD1d by mouse Vα14 and human Vα24 natural killer T (NKT) cells, and plays an important role in regulating NKT cell responses during infections, cancer and autoimmunity (47, 57, 106–108). In addition, a unique Galα1-2GalCer was found in BMDC as well, which can be processed to GalCer for presentation to NKT cells (109). Based on the upregulation of globosides during the differentiation of macrophages, moDCs and BMDCs, globosides function as markers of differentiation (57).

### Lymphocytes

Lymphocytes include T cells, B cells, and natural killer (NK) cells (Figure 2), which are the main adaptive and innate immune effector cells. GSL expression in B and T cells has been widely studied during differentiation, maturation, and immune responses.

#### B Cells

After antigen exposure, B cells can differentiate into plasma cells secreting antibodies to clear antigen-bearing entities. Human pre-B cells have a similar GSL-profile to cells of myeloid origin. Human B cells mainly express GM3, but also more complex gangliosides such as GM1, GD1a, GD1b, and GT1 (32, 58, 63). In addition, asialo GM1 and asialo GM2 are expressed in minor amounts (61). Notably, ganglioside GD3 and its *O*-acetylated variants, 7-*O*-GD3 and 9-*O*-GD3 (CD60b and CD60c, respectively), have been described to be expressed on B cells (and also T cells) although the expression levels vary (12, 50, 72). Some of these studies propose an involvement of *O*-acetylated gangliosides in lymphocyte activation processes. Mouse B cells show an even higher expression of the gangliosides GM1 and GM3 and their derivatives compared to human B cells. Interestingly, whereas humans are incapable of synthesizing *N*-glycolylneuraminic acid (NeuGc), gangliosides GM1 and GM3 modified with this sugar are present on mouse B cells. Importantly, the CD22 ligand Neu5Acα2-6Gal-, also known as CD75 (Figure 1B), was identified as a major B lymphocyte epitope (95). Additionally, rat B cells lowly expressed Galα1-3(F(2))ASGM1 and some unique extended GM1b structures, which contain the GM1b core extended with LacNAc unit(s), including Galα1-3LacNAc-GM1, Galα1-3(LacNAc)_2_-GM1, and S(3)LacNAc-GM1 (110).

Both human and murine B cells express GalCer, GlcCer, LacCer, and globosides, but only immature B cells contain (neo)lacto-series GSLs since activated B cells lack expression of the Lc3 synthase B3GNT5 (23, 63, 65, 66). Human peripheral B cells contain relatively large amounts of more complex globosides which are nearly absent in tonsillar B lymphocytes (32, 62). Importantly, Gb3 (CD77) was initially found to be specifically expressed by germinal center B cells (60, 111). However, it was later identified that not all germinal center B cells express Gb3 (112). In contrast to peripheral and germinal center B cells, GlcCer, and LacCer comprise the largest portion of GSLs in tonsillar B lymphocytes. In addition, Gb3 expression increased 10-fold in a bovine B cell lymphoma cell line after stimulation with different mitogens, suggesting that B cells actively regulate surface expression of Gb3 (113).

Human B cell differentiation and activation are accompanied by sequential regulation of GSL expression via modulation of the corresponding GTs (61, 63, 114). GM3 synthase B4GALNT1 is differentially activated from the pre-B cell stage to the terminally differentiated myeloma (plasma)cells, and GM2 synthase B4GALT has a high activity in lymphoblastoid cell lines and terminally differentiated myeloma cells only. Lc3 synthase B3GNT5 shows a high activity in pro- and pre-B cells, initializing the synthesis of (neo)lacto-series GSLs. But, (neo)lacto-series synthesis is shut down in more differentiated cells. For the expression of globosides, Gb3 synthase A4GALT and Gb4 synthase B3GALNT are only activated in the late stages of B cell differentiation (114). These results explain the stage-dependent expression of GSLs like Gb3, Gb4, GM2, and GM3, suggesting functional roles of GSLs during B cell maturation (63).

#### T Cells

T cells are the effector cells of adaptive immunity through the production of various cytokines and the activation-induced cell death. Variations in GSL expression have been related to T cell subtype, activation, differentiation, and function (66, 67). Human T cells express both GM1 and GM3, which are clustered in GEMs and thought to be involved in T cell activation (66). Besides these two gangliosides, also minor levels of other gangliosides (GD1a, GD1b, GT1b etc.) have been detected (18, 115, 116). During interleukin-2 (IL-2) stimulation, CD8^+^ T cells, more than CD4^+^ T cells, upregulate GM1 expression (117, 118). In contrast, naïve CD4^+^ T cells stimulated with anti-CD3/CD28 show increased expression of ST8SIA1, driving GD3 expression (119). Similar to B cells, *O*-acetylated variants of the ganglioside GD3 have been described to be expressed by human T cells (10, 12, 13, 50). Desialylation of GSLs was also apparent in T cells, since the sialidases NEU1 and NEU3 are 2- to 3-fold upregulated upon T cell receptor (TCR) ligation of both CD4^+^ and CD8^+^ T cells. Interestingly, inhibition of these sialidases resulted in a greater amount of cell surface sialic acids, but also a reduced IFN-γ secretion upon activation of T cells (120, 121). These data indicate that T cell effector function can be modulated by sialic acid bearing GSLs in T cells.

Similar to human T cells, murine T cells express GM3, GM1a, GM1b, GD1b, GD1c, GD3, asialo GM1, and extended GM1b series. Compared to CD8^+^ T cells, murine CD4^+^ T cells express higher level of ST3GAL5 to synthesize *a*- and *b*-series gangliosides (GM1a and GD1b). In contrast, CD8^+^ T cells express more B4GALNT1, resulting in higher levels of *o*-series gangliosides (asialo GM1, GM1b, GalNAcGM1b, and extended-GM1b) (66, 68, 70, 71, 122–126). Although these studies show that stimulation of T cells correlates with elongation of a common GM1b precursor structure, it is as yet unclear how such GSLs contribute to T cell physiology.

The total amount of gangliosides per cell was found to be about 10-fold higher in mature T cells than in thymocytes. This increased level of ganglioside expression mainly resulted from the upregulation of GM1 subclasses and *o*-series gangliosides (GalNAcGM1b and extended-GM1b) in T cells whereas GD1b is downregulated (70, 71). This distinct expression of gangliosides between murine thymocytes and mature T cells suggest a stage and type-dependent expression of gangliosides, similar to B cells (71). Notably, whereas GD1c is highly expressed in both thymocytes and CD4^+^ T cells, CD8^+^ T cells downregulate its expression (68, 116, 127). Similarly, GM1a is present on both thymocytes and CD4^+^ T cells, while only trace amounts are found in CD8^+^ T cells (70). Compared to the human T cells, activated murine CD8^+^ T cells also upregulate the sialidase NEU3 and downregulate NEU1 (128). In addition, some unique modified GM1 series, including Galα1-3LacNAc-GM1, Galα1-3(LacNAc)_2_-GM1, and S(3)LacNAc-GM1 were found in rat thymocytes (110, 129).

With respect to neutral GSLs, both human and murine T cells express GlcCer, LacCer, asialo GM1, globosides, and (neo)lacto-series (57, 58, 67, 71). In murine and rat T cells, quantification of neutral GSLs has revealed that the amount of neutral GSLs was higher in peripheral T cells compared to thymocytes. The major neutral GSLs in thymocytes are globosides while asialo GM1 is the most abundant neutral GSL in mature T cells (58, 69, 130, 131). In addition, some unique neutral GSLs, such as Galα1-3(F(2))ASGM1, have been detected in rat thymocytes (110). The presence of isoGb3 on T cells was recently described, which is recognized by both mouse and human NKT cells when presented by CD1d (57). However, the relevance of this GSL for NKT cells remains to be elucidated since mice that lack the isoGb3 synthesis machinery show a normal phenotype and function (47).

#### NK Cells

NK cells develop in bone marrow and account for up to 15% of peripheral blood mononuclear cells. NK cell activity is unleashed by a loss of inhibitory signaling of their receptors that recognize MHC class I on a target's cell surface, which often is the case on infected or malignant cells.

To date, the GSL expression on NK cells has not been well-studied. In contrast to NK cell precursors, mature NK cells express asialo GM1 (69, 73, 74, 80). Besides asialo GM1, NK cells in mice have been reported to express GM1 at a relatively high level compared to splenic T cells (69). The ganglioside 7-*O*-acetyl GD3 was found at medium levels in 16% of the CD16^+^ NK cells (50, 72).

### Considerations Concerning GSL Expression Analyses

Many studies have contributed to the current knowledge of GSL expression in immune cells, during development, maturation, or activation. Still, information on GSL subtype expression in several immune cell subsets is incomplete (Table 1 and Figure 2) and in many cases lack structural details, often due to the limitations of the analytical tools employed. Incomplete structural information poses a challenge in understanding expression, regulation, and function of GSLs in immune cells. Thus, further in depth structural studies are pivotal as a basis for functional investigations.

It is clear though that the subtypes of GSLs are very differentially expressed throughout the immune system, suggesting that GSLs not just constitute a structural requirement for membrane integrity of immune cells but rather play specific roles in their function. For example, (neo)lacto-series GSLs are highly expressed by neutrophils, but not their progenitor cells, suggesting a specific role in neutrophil mediated immunity. This may relate to pathogen recognition through an interaction of neolacto glycans with pathogen-expressed proteins (132). On the other hand, it is curious that the expression of some GSLs by human immune cells significantly differs from their murine counterparts. Does this mean that GSLs are functionally dispensable or at least replaceable? A few functions of GSLs have been identified and will be discussed below. Furthermore, GSL expression alterations in response to cytokines and other modulators have also been observed, suggesting an intricate regulation of synthesis and degradation which will be discussed in the next chapter.

## Regulation of GSL Expression in Immune Cells

**Figure 3:**
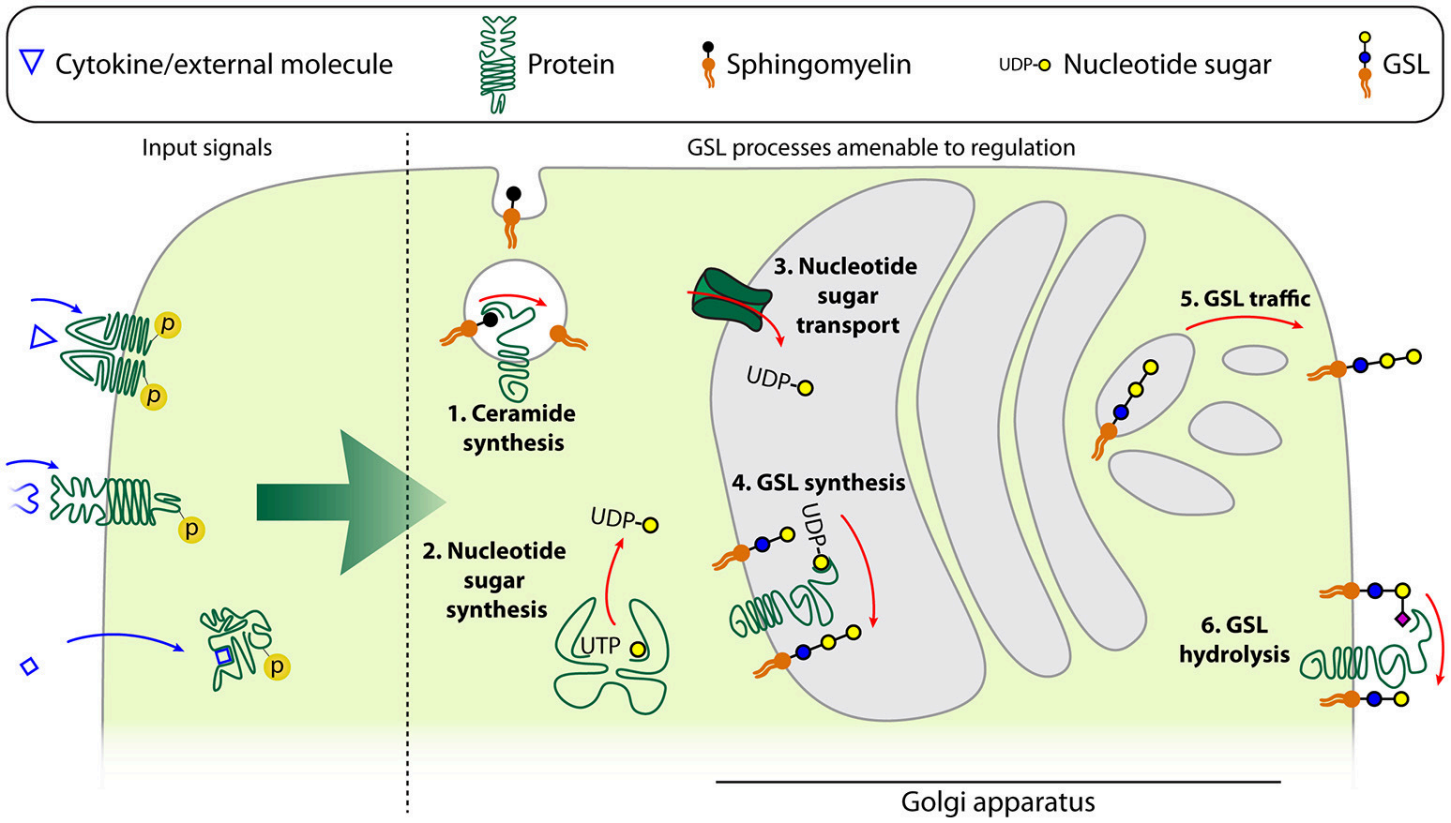
Schematic model of the different levels of GSL regulation. Input signals described to be able to affect the GSL repertoire of a cell are either cytokines, other ligands with membrane-bound receptors or ligands with intracellular receptors. These affect the GSL repertoire by changing the expression or activity of ceramide synthases such as acidic sphingomyelinase (1), nucleotide sugar synthases in the cytoplasm (2), nucleotide sugar transporters which transport the nucleotide sugars into the Golgi apparatus (3), glycosyltransferases (4), trafficking of the GSLs from the Golgi apparatus to the plasma membrane (5), or glycosidases (6).

Differentiation and activation of immune cells leads to alterations in the GSL repertoire, likely through modulation of the expression of GTs, glycosidases, glycan precursor synthesizing enzymes, and nucleotide sugar transporters (Figure 3) (14–16). Although these processes are well-documented, little information is available on the regulation of GSL expression in immune cells specifically. Nevertheless, the GSL regulation in the context of immune cell differentiation and activation as described in Biosynthesis and Expression of GSLs in Naïve and Differentiated Immune Cells, is often regulated by well-known signals, such as cytokines. We will now further focus on the molecular details of such external signals on the regulation of GSL synthesis and expression in immune cells.

### Regulation of GSL Expression by Cytokines

It is yet largely unclear what the intracellular switches and master regulators of GSL expression are. Knowledge of cytokine-induced signaling cascades, whether or not in the context of differentiation or activation, is important to understand GSL regulation and may provide opportunities for the design of intervention strategies. Up to now, regulation of GSL expression on immune cells has mainly been studied by addition of key cytokines such as interleukins, interferon-α (IFN-α), and tumor necrosis factor-α (TNF-α) (Figure 3).

IL-4 and especially IL-6 induce expression of Fo GSLs at early stages of mouse BMDM culture, but neither could promote further Fo GSL expression once the intrinsic maximum of these cells had been reached (104, 105, 133). The mechanism of these IL-4 and IL-6 regulated differences in GSL composition is still unclear. One option may be that these interleukins coordinate GSL synthesis through modulation of the nucleotide sugar metabolism. IL-4 and IL-13 have the ability to upregulate the levels of UDP-GlcNAc which is a key nucleotide sugar donor for GSL synthesis. The increased activity of corresponding transcriptional enzymes involved in the production of these intermediates (e.g., Enpp1, Pgm1) was reported for IL-4 activated M2 polarized macrophages as well, and was not observed in IFN-γ and toll-like receptor-induced M1 macrophage polarization (134). An alternative mechanism of GSL regulation was provided by overexpression of IL-3 in mouse NFS60-17 cells, which leads to the specific synthesis of GD1a (114, 123, 135). This change in GSL expression is caused by increased GM3 synthase levels, since other GTs involved in GD1a synthesis were not significantly altered by IL-3 expression. Thus, regulation of GT expression can result in a shift in the GSL repertoire, in this case from *o*-series to *a*- and *o*-series gangliosides (Figure 3). IFN-α induces more significant alterations in GSL biosynthesis in mouse B cells compared to other cytokines, including IL-6 and IL-10. In particular, GlcCer, LacCer, and Gb3 are significantly upregulated (65). These changes were attributed to the enhanced expression of UGCG and A4GALT. IFN-α also represses α-galactosidase that catalyzes the degradation of Gb3 further contributing to Gb3 accumulation (65). The effect of TNF-α on GSL expression has been described in several studies. TNF-α binding to its cognate receptor TNFR1 has been reported to enhance ceramide production by upregulating the acidic sphingomyelinase, a ceramide generating enzyme (136, 137). Furthermore, TNF-α increased Gb3, GM2, and GM3 through increased transcription of their specific synthases (134, 138–140). TNF-α also mediated upregulation of GM2 in tumor cells and accelerated tumor-induced T cell apoptosis and immune dysfunction. Furthermore, TNF-α was found to activate sialidases through p38 mitogen-activated protein kinase in lipopolysaccharide(LPS)-stimulated human monocytes, suggesting that TNF-α-induced p38 activation may regulate GSL expression (141).

### Regulation of GSL Expression by Other External Signals

Not only cytokines but also other factors have been observed to alter GSL expression. The presence of high-affinity FcεRI is suggested to contribute to the expression of gangliosides. FcεRI positive HMC-1 cells expressed 3-fold higher levels of GM3 compared to the FcεRI negative counterparts. Furthermore, detectable amounts of the gangliosides GM2, GM1, and GD1a were found only in the FcεRI positive HMC-1 cells, with a corresponding increase of mRNA for GalNTs in the presence of the FcεRI. These findings suggest that FcεRI signaling enhances ganglioside production (25). Similarly, TCR stimulation on naïve CD8^+^ T cells upregulated GM1 expression, which is crucial for responding to self-MHC ligands and IL-2 (117). GM1 levels declined after cell transfer to MHC-I^low^ (Tap^−/−^) mice, indicating that maintenance of GM1 expression required continuous TCR-MHC-I interaction. By contrast, CD4^+^ T cells expressed low amounts of GM1 and were unresponsive to IL-2 (117). In addition, both NEU1 and NEU3 mRNAs were significantly induced in human T cells by TCR stimulation, potentially leading to a decrease of sialylated GSLs (Figure 3) (120). Wang et al. further revealed that NEU3 is expressed as a major isoform in activated cells. Transcription of NEU expression in T cells is enhanced by FLI1, whose activity is potentially driven by TCR stimulation. Genetic reduction of FLI1 expression in T cells thus decreased NEU1 and NEU3 levels but also overall GSL expression. However, the mechanism by which FLI1 influences GSL expression is not clear yet (118). GSL levels on CD4^+^ T cells can also be boosted by stimulation with synthetic liver X receptor (LXR), which signals through the nuclear receptor LXRβ. Stimulation of LXR is known to directly control expression of NPC1 and NPC2 proteins, which regulate cellular GSL transport and recycling (Figure 3). Therefore, an elevated LacCer, Gb3, and GM1 expression in CD4^+^ T cells with highly expressed LXRβ was achieved, which associated with accelerated and sustained GSL internalization and recycling dynamics. Interestingly, this enhanced GSL expression is not correlated with changes in synthase expression but rather associated with the intracellular accumulation and accelerated trafficking of GSLs (67). Yet another GSL modulating stimulus is heparin, which modulates the expression of GSLs in lymphocytes activated by IL-2. Heparin treatment induces downregulation of certain GSLs, including GM1, GD1a, LacCer, asialo GM1, and asialo GM2, whereas globoside and Fo antigen levels are elevated. These changes were attributed to heparin-mediated inhibition of α2-3 sialyltransferase and a β1-3 galactosyltransferase, possibly via heparin-binding domains (142).

### Considerations on Regulation of GSL Expression

GSL expression is highly controlled at multiple levels, such as the availability of nucleotide sugars and glycosyltransferases (Figure 3). Our understanding of how the GSL synthesis pathway is regulated in specific immune cells needs be improved. The fact that the physiological role of most immune cells is known will then provide opportunities to unravel molecular functions of specific GSLs in these cells. In addition, various laboratories have identified environmental factors that manipulate the GSL repertoire by seizing on components of the GSL synthesis pathway. The limited number of papers describing such regulation of GSL synthesis clearly indicates that this is an underexposed field. Moreover, the available data seems to be biased toward the more well-known soluble proteins. We expect many more GSL regulatory factors to exist that are not yet linked to GSL synthesis. The identification of such GSL modulatory processes may have implications for GSL manipulation in research and potentially even in clinical contexts.

## Functions of GSLs on Immune Cells

### Organization of Membrane Microdomains

**Figure 4:**
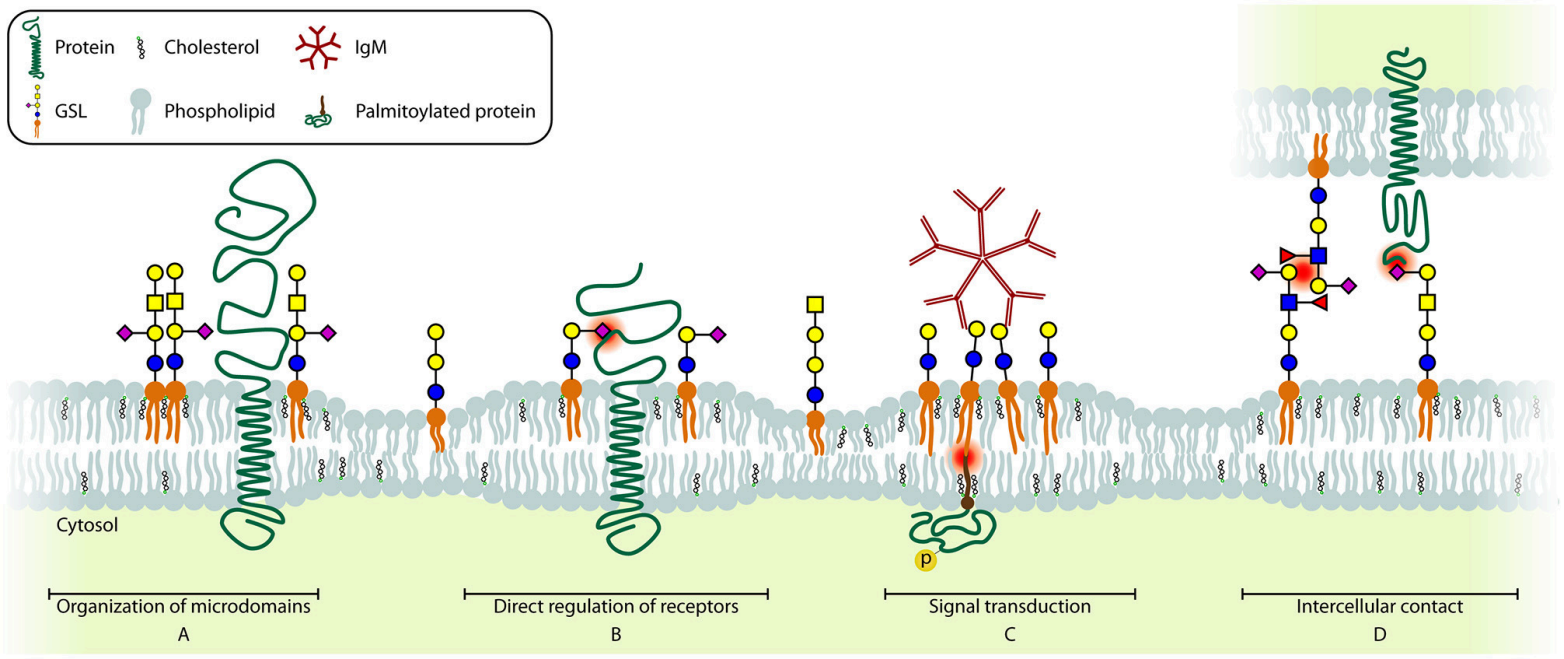
Schematic model of the different GSL functions. Essential glycan-glycan, protein-glycan, and lipid interactions are highlighted (red dot). **(A)** GSLs are involved in including and (not shown) excluding proteins from microdomains. **(B)** Several receptors can be directly regulated by GSLs present in the cell membrane. **(C)** Crosslinking of several GSLs can induce signaling across the membrane. **(D)** GSLs can interact with glycans (CCI, left) or with proteins (PCI, right) on other cells, contributing to cell-cell recognition and adhesion.

GSLs are mainly known for their role in membrane organization which is a dynamic process, especially during activation and differentiation of immune cells. In resting immune cells, GEMs (Figure 4A) are suggested to be unstable and small in size. Immune cell activation triggers a change in localization of receptors and signal transducers, in many cases to or from GEMs, which is required to bring receptors and signal transducers in close proximity to enable signaling (143, 144). The best described example in T cells is the activation-induced recruitment of the TCR/CD3 complex to GM1 GEMs together with downstream signaling molecules Lck, SLP-76, and palmitoylated LAT. At the same time, the phosphatase CD45 is excluded from GEMs, further increasing the sensitivity of the TCR (145–151). Additionally, the IL2Rβ is recruited to GM1 GEMs upon stimulation, which is required for its signaling (117). Interestingly, when GM1 GEMs were crosslinked by CTB and anti-CTB antibodies, TCR-like signaling was observed, suggesting that multiple signaling molecules are brought together by crosslinking multiple GM1 GEMs, which indicates a diversity in GM1 GEMs content in different plasma membrane patches (148). General disruption of GEMs in T cells results in a lack of receptor recruitment and exclusion from the immunological synapse, which causes desensitization for ligands and greatly reduced or absent T cell activation. Interestingly, no difference in T cell development has been observed in mice with a T cell specific deletion of UGCG. However, no functional characterization was performed on these T cells other than PMA/ionomycin stimulation, which bypasses signaling from the membrane. In contrast, the development of invariant NKT cells that recognize CD1d-restricted antigens was found to be impaired in these mice (152).

Once B cells encounter an antigen, caveolin-1 recruits the IgM BCR to GM1 GEMs (153–155). The lack of caveolin-1 results in impaired BCR signaling which results in decreased receptor editing and ultimately autoimmune B cells (155). Also MHC-II molecules on B cells require clustering to GM1 GEMs in order to efficiently trigger CD4^+^ T cell help at low ligand densities (156, 157). Additionally, B cells in B3GNT5^−/−^ mice, which lack (neo)lacto-series GSLs, display alterations in the structure of GM1 GEMs containing BCR, CD19, and Lyn, resulting in increased antigen sensitivity. Consequently these B cells are also more prone to generate autoreactive antibodies (158).

Thus, in both T and B cells, GM1 is part of GEMs that have a crucial role during activation of these cells. The fact that other GSLs expressed by these cells (see section Biosynthesis and Expression of GSLs in Naïve and Differentiated Immune Cells) have not been investigated in this context is likely due to a lack of detection and visualization methods. Importantly, the plasma membrane may contain a large diversity of domains or GEMs with a slightly different (glycosphingo)lipid and protein content as proposed in the revised Fluid-Mosaic Membrane Model (144). Each domain with physically distinct properties may have a specific function, also in immune cells (144). Techniques to visualize these nanometer-domains without introducing artifacts are still scarce. While detection of GM1 by cholera toxin CTB is a broadly accepted method, probes that are considered specific for other GSLs are less well-established, introducing a strong bias toward GM1 GEMs investigations. Therefore, the function of other GSL containing membrane domains and their role in membrane organization is still largely unclear.

### Direct Regulation of Immune Receptors

A second function of GSLs is their direct regulation of surface protein function (Figure 4B). One of the best-described interactions between GSLs and proteins is the interaction between insulin receptor and GM3. Insulin receptor binds the acidic GM3 through a basic lysine residue (K944) resulting in inhibition of insulin-induced signaling. Thus, upregulation of GM3, for example after TNF-α stimulation, results in insulin resistance (139, 159). Similarly, autophosphorylation of the Epidermal Growth Factor Receptor (EGFR) in the absence of EGF is prevented by binding of GM3 via a lysine residue (K642) (160).

On immune cells, only few GSL-receptor interactions have been reported, often with limited molecular details. Upon activation of the Toll-Like Receptor 4-Myeloid Differentiation factor 2 (TLR4-MD2) with LPS, Gb4 synthesis is upregulated in mouse endothelial cells. Gb4 can bind the TLR4-MD2 complex to desensitize the LPS-activated signaling pathway thus representing a negative feedback loop (161). Since Gb4 and the TLR4-MD2 complex are also expressed on early human myeloid cells and mature monocytes (23), one may speculate that a similar regulation applies to human immune cells. Interestingly, GlcCer on the membrane of macrophages is essential for efficient LPS-induced TLR4-MD2 signaling since inhibition of GSL-synthesis prior to incubation with LPS significantly reduced cytokine release. *In silico* simulations to explain these observations suggest that GlcCer induces a conformational change of TLR4 thereby enhancing the interaction between TLR4 and the intracellular signaling molecule Mal (52).

In T cells, CD4 interacts with GM1, and additional GM1 incorporation into the membrane results in masking of some CD4 epitopes for antibodies and a subsequent internalization of CD4 molecules, with the underlying mechanisms being unknown. Additionally, GM1 binds PI3K whereas GM3 binds LCK. In order to get successful T cell activation LFA-1 links CD4 and PI3K to LCK by binding both GM1 and GM3 (162, 163).

GSLs are also important for strengthening protein-protein interactions in tetraspanin-rich microdomains. An example of the stabilizing function of GSLs is the enhanced binding of the tetraspanins CD9 and CD82 to integrins in the presence of GM3 and GM1, respectively (164, 165). Thus, GSLs may impact integrin mediated immune cell migration (166). Additionally, loss of functional tetraspanin-rich microdomains results in uncontrolled receptor activity, such as uncontrolled activation of the MET receptor tyrosine kinase and decreased EGFR sensitivity (165, 167). CD82 expression also correlates with increased GM1 and GD1a levels on the cell surface, suggesting an interplay between GSLs and tetraspanin expression either by increasing GSL synthesis or by extending the half-life of GSLs on the plasma membrane (168).

Since CD19 shares amino acid sequences with the Gb3 binding domain of the *E. coli* produced verotoxin, the Daudi B cell line was modified to lack Gb3, which impaired CD19 surface expression. However, the mechanism was not elucidated and since only a subpopulation of germinal center B cells express Gb3 while CD19 is expressed on all B cells, the finding may be an artifact of the cell system that was used (112, 169). Using the same approach MHC-II was identified as another protein that contained a possible Gb3 binding domain, which could be relevant in for example germinal center reactions of B cells, but also for other professional antigen presenting cells such as macrophages and DCs which also express considerable amounts of Gb3 (see section Biosynthesis and Expression of GSLs in Naïve and Differentiated Immune Cells). Unfortunately, no binding data are available for the MHC-II-Gb3 interaction, thus the functionality of these domains is still unclear (170).

Activation of Notch by its ligand Delta-like 1 (Dll1) is dependent on binding of Dll1 to LacCer. Either mutating the LacCer binding site of Dll1 or inhibiting GSL synthesis impairs the capacity of Dll1 to activate Notch (171). This may be relevant during T cell development, where Notch signaling plays a major role (172).

The internalization route of Fas receptor upon ligation with Fas ligand is determined by its interaction with LacCer or Gb3 which results in an endocytotic pathway leading to apoptosis, while the GSL-independent route induces proliferation and differentiation (173). Expression of Gb3 by B cells (112) during the germinal center reaction may support the apoptotic events required for B cell selection.

Besides direct interactions between GSLs and proteins described above, there are also reports on interactions between *N*-glycans and GSLs. The ganglioside GT1b can interact with mannose residues on the *N*-glycan of the α5-integrin, thereby inhibiting integrin-fibronectin interaction (174). Regulation of integrin activity by GT1b may play a role in T cell development, where α5β1 integrin signaling plays a role in T cell selection (175, 176).

### GSLs as Signal Transducers

Direct interaction of GSLs with surface receptors may thus have profound impact on signaling events. But GSLs can also transduce signals across the membrane themselves (Figure 4C). Crosslinking GSLs by multivalent binders such as bacterial toxins CTB and Shiga Toxin (ST), or alternatively IgM antibodies, has been found to increase intracellular calcium levels that in turn activate Syk (177, 178). This influx of calcium ions upon GM1 crosslinking on the cell surface may be through modulation of L-type calcium channels. Additionally some GSLs regulate intracellular calcium levels by affecting the function of the calcium-dependent messenger protein calmodulin (179, 180). The result of Gb3 crosslinking using ST or anti-Gb3 mAbs in germinal center B cells induces recruitment of Lyn/Syk and the BCR and subsequent internalization of the complex leading to apoptosis (181). Interestingly, the pathways leading to apoptosis differ between ST or anti-Gb3 mediated crosslinking of Gb3 (182–185). Similarly, crosslinking of GM1^+^ patches in T cells using crosslinked CTB induces LCK-dependent TCR-like signaling (148). Interestingly, crosslinking of GM1^+^ patches by the *E. coli* heat-labile enterotoxin B induces apoptosis in CD8^+^ T cells specifically (186). However, there are some doubts on the specificity of these two toxins, which may explain differences in results obtained.

In neutrophils, the kinase Lyn is associated with LacCer enriched microdomains. Crosslinking of these microdomains by anti-LacCer IgM antibodies induces Lyn activation and ultimately leads to superoxide production (39). This signal transduction from LacCer molecules to the palmitoylated form of Lyn is dependent on the length of the fatty acid chain of the GSLs; Lyn is only activated when the fatty acid chain contains 24 carbon atoms and not with shorter fatty acids of 22 or 16 carbon atoms, suggesting that the signal is transmitted within the lipid bilayer relying on specific interactions of the lipid tails (187, 188). Although the length of the fatty acid chain also influences the general membrane organization and association with proteins which is not addressed yet, a similar association has been described for Lyn and c-Src with photoactivatable GD1b in rat cerebral granule cells (189).

### Intercellular GSL Functions

There are two mechanisms by which cells interact with GSLs on other cells; via protein-carbohydrate interaction (PCI), and via carbohydrate-carbohydrate interaction (CCI) (Figure 4D). Proteins known to engage in PCI are called lectins, and human lectins may be grouped into three major classes; (1) selectins, that typically bind glycans that are both sialylated and fucosylated, (2) siglecs, which bind sialylated glycans, and (3) galectins, that bind glycans with a terminal galactose. The function of these lectins differ per cell type, with selectins being the major mediators of cell-cell adhesion, particularly between activated endothelial cells and leukocytes. Siglecs specifically interact with sialic acids and are mainly found on hematopoietic cells. Galectins, on the other hand, often bind terminal galactoses and can modulate cell growth, apoptosis, differentiation, and migration (190).

CD83 is an I-type lectin adhesion receptor that is mainly expressed by mature dendritic cells but is also found on activated B and T cells. CD83 interacts with sialic acids on monocytes and activated CD8^+^ T cells and is required for efficient T cell activation (191). Although the ligand for CD83 was identified as a glycan carried by a glycoprotein on the T cell line HPB-ALL, the authors do not rule out the possibility of ligands carried by GSLs (192).

The sialic acid binding receptor on B cells, CD22 or siglec-2, recognizes α2,6-linked sialic acids that are predominantly expressed in eukaryotes. When the B cell is in an inactive state, CD22 is associated with sialic acids on the B cell surface. However, once the B cell becomes activated, the CD22 is unmasked, and can engage in *trans*-interactions with sialic acids on other cells which induces inhibitory signaling (193, 194). NK cell activation may be controlled by siglec-7 in a similar manner (195, 196). The current hypothesis is that these interactions prevent activation of auto-reactive B and NK cells (197).

Cell-cell interaction in the immune system is critical at sites of inflammation. Inflammation-mediated activation of endothelial cells upregulates selectins like E-selectin in order to recruit immune cells (198). The ligand for E-selectin on neutrophils is a GSL that contains poly-LacNAc repeats with at least two fucose residues and a terminal sialic acid, but E-selectin may also bind GSLs and glycoproteins containing the sialyl-Le^x^ motif (Figure 1B). This interaction is of low affinity and induces typical neutrophil rolling on the endothelium, which is required for transmigration afterwards (199).

CCIs are studied to a lesser extent compared to PCIs. They are involved in early embryogenesis, where the compaction of the embryo is dependent on Le^x^ structures [for review, see (200)]. Additional reports on CCI describe the interaction between GM3 or Gb4 and asialo GM2 (201). Although a single CCI is generally of very low binding affinity, the carbohydrates may be so prevalent that they may act as a zipper to mediate strong cell-cell adhesion, comparable to CPI or even protein-protein interaction (200, 202).

Although still poorly understood, B cells communicate by forming nanotubes in certain differentiation stages which correlate with expression of GM1 and GM3. The formation of these nanotubes was inhibited by methyl-β-cyclodextrin induced cholesterol depletion, which destroys the integrity of GEMs. Furthermore, only cells with high levels of raftophilic sphingomyelin and phosphatidylcholine generated nanotubes. Thus, the formation of these nanotubes depends on functional GEMs which is possibly related to their GSL contents (203).

### Considerations on Molecular Functions of GSLs

GSLs clearly play a role in immunological processes involving cell-cell recognition, adhesion, and communication. However, most of the studies merely provide evidence that certain GSLs are required or sufficient for a particular process, while the exact molecular role of such GSLs remains to be identified for most of these processes. Such mechanistic studies are sparse for a reason, because molecular evidence is often hard to obtain with the current tools. Furthermore, the studies are still limited to a few specific GSLs and do not cover all GSL subtypes. For example, (neo)lacto-series GSLs have largely been neglected in investigations. The relatively recent generation of B3GNT5 knockout cancer cell lines and mice are important initiatives to extend our knowledge on the physiological role of these elusive GSLs (158, 204). Thus, many aspects of GSL functions are still unclear and require further in depth investigations.

## Relations Between GSLs and Immunity in Disease

Congenital diseases, infections, and cancer showcase aberrant GSL expression, which provides opportunities to gain new insights in (dys)regulation and functions of GSLs. Such knowledge may provide new targets for therapeutic intervention, of which the most recent developments are described in section Targeting GSLs: Opportunities for Treatment.

### Gaucher Disease

Patients with Gaucher disease lack the enzyme glucosylceramidase, which is required for the breakdown of GlcCer. Besides neuronal abnormalities this disease is characterized by the presence of large “Gaucher cells” which are macrophages with accumulated GlcCer in lysosomes that concentrate in the spleen and bone marrow. The formation of splenic Gaucher cells is enhanced by rapid splenic clearance of defective red blood cells by macrophages (205). Patients suffering from Gaucher disease are treated either with enzyme replacement therapy or with substrate reduction therapy which consists of the administration of UGCG inhibitors such as *N*-butyl-deoxynojirimycin (Miglustat) (205, 206).

### Infection

Various pathogens dysregulate the cellular GSL metabolism, leading to different compositions of the cell surface GSL repertoire. The p40^tax^ protein encoded by the human T cell lymphotropic virus, can induce GD2 expression by upregulating B4GALNT1, which is normally not expressed in T cells (207). Similarly, it was shown that cytomegalovirus (CMV) induces enhanced synthesis of GSLs, of which specifically (neo)lacto-series remain expressed long after initial infection (208, 209). Additionally, herpes simplex virus alters gene expression of a variety of GTs. The significance of these changes still need to be addressed since the authors could not detect major differences in the profile or total amount of GSLs after infection (210). A potential reason for such dysregulation may be to escape from detection and elimination by the immune system.

Several infectious pathogens and toxins are well-known to use GSLs as cellular entry receptor. Next to CD4, HIV can infect cells through Gb3 and possibly also GM3. *Shigella* bacteria target only activated CD4^+^ T cells likely through their GM1 and GM3 expression which was inhibited by exogenously added LPS, suggesting a direct interaction between LPS and the gangliosides (211). This would imply that also other gram-negative bacteria may enter host cells through binding of their gangliosides (212).

A variety of bacterial toxins have been described to target GSLs using their binding subunit (B subunit) in order to bring their enzymatically active subunit (A subunit) inside the cell. In 1973, one of the best known toxins, cholera toxin, was described to bind GM1 (213). Although generally used as a marker for GM1, CTB can bind asialo GM1, Fuc-GM1, GD1a, GD1b, GT1b, GM2, GM3, and also to Le^x^ on glycoproteins although usually with lower affinity. Similarly, it was long thought that enterotoxin B was GM1-specific, until it was shown to cross-react with asialo GM1, GD1b, LacCer, and several galactoproteins (214–216). The B subunit of shiga toxins (STb) and verotoxins associate with Gb3, although all bind Gb3 in a slightly different way (217). Since STb binding to Gb3 induces endocytosis and Gb3 is present on DCs, some research has been devoted to exploiting STb for tumor vaccination (218). However, STb elicited a cytotoxic effect through binding of an *N*-glycan on HeLa cells, suggesting this strategy may have serious side-effects when applied in humans (219). The toxic effects of tetanus toxin and botulinum toxin were greatly reduced in B4GALNT1 (GD2-synthase) deficient mice, suggesting their natural ligands are at least partly complex gangliosides (220). Confirming these findings, type A botulinum progenitor toxin bound asialo GM1, nLc4 and *N*-glycans containing a terminal Galβ1-4GlcNAc (221). Despite these health risks, the physiological function of specific GSL structures was apparently too critical to be efficiently counterselected against during human evolution. Although GSLs are essential during embryonic development, this may also partially be due to the versatile roles of GSLs in immunity.

Finally, several bacteria have the capacity to bind GSLs but it is currently unclear what the pathophysiological reason is for this phenomenon. *Helicobacter pylori*, a microaerophilic organism that can cause severe gastritis, binds to sialic acid-containing GSLs on neutrophils, thereby activating the neutrophil to produce reactive oxygen species (222, 223). Interestingly, neutrophils can phagocytose the bacteria but it seems able to escape the immune cell and cope with the immune response (222, 224). *Neisseria* bacteria, mostly known for their genera *meningitides* and *gonorrhoeae*, are also capable of binding GSLs, although it differs per strain which GSLs they adhere to. *N. subflava* binds sialylated GSLs on erythrocytes by its adhesin Sia1 (225) whereas *N. gonorrhoeae* has an adhesin binding LacCer and asialo GM1 (226). *N. meningitides* binds a wider array of GSLs; LacCer, asialo GM2, asialo GM1, nLc4 but also sialylated nLc6 (227). Additionally, phagocytosis of *N. meningitidis* by neutrophils appears to depend on their expression of (neo)lacto-series GSLs since it is blocked by the LacNAc-Gal-binding antibody 1B2 (228).

The importance of GEMs for the phagocytosis of yeast, such as *Cryptococcus neoformans*, by macrophages has been well-defined since disruption of GEMs using methyl-β-cyclodextrin decreases internalization (229). However, Jimenez-Lucho et al. have shown specific binding of *C. neoformans, Candidia albicans*, and other fungi to LacCer, suggesting indeed a role of these GSLs as adhesion receptors for yeast (230). This was confirmed by the identification of an interaction between the bacterial and fungal cell wall polysaccharide β-glucan and LacCer on neutrophils, which triggers superoxide production and CD11b/CD18-mediated phagocytosis of the pathogen (231). These examples indicate potential pathways for different pathogens to be captured by phagocytes, which play an important role in the antimicrobial defense. Moreover, the specific GSL repertoire of neutrophils may allow for improved detection of bacteria, or other pathogens, and possibly contribute to fight infections.

### Cancer

Tumors often express high levels of GSLs, which interferes with the killing capacity of the immune system. These high levels of GSLs result, either via an active or passive process, in high concentrations of free GSLs in the tumor microenvironment. For some tumors, such as neuroblastoma, the plasma concentration of tumor-derived GSLs was 50 times elevated as compared to the same patients after treatment or healthy controls (232, 233). Multiple modes of action have been described for the immunosuppressive characteristics of free GSLs.

A portion of T cells isolated from renal cell carcinoma were found to be GM2 positive, while lacking the machinery for GM2 synthesis, suggesting the T cells adopted the GM2 from the tumor microenvironment. These T cells exhibited increased rates of apoptosis compared to their GM2 negative counterparts (234). In addition, *ex-vivo* T cells treated with renal cell carcinoma-derived gangliosides also show a decrease in NFκB signaling (235). T cells incubated with exogenous GD1a lose cytotoxicity since polarization and exocytosis of lytic granules is inhibited, we speculate this may also be due to incorporation of soluble gangliosides in the plasma membrane, disrupting the organization required for proper T cell function (236). Additionally, CD4^+^ T cells cultured in the presence of GT1b led to a shift from an IFN-γ secreting type-1 phenotype to an IL-4 producing type-2 phenotype (237). Finally, various individual brain-derived gangliosides inhibit T cell proliferation possibly through competing for the IL-2 binding place on the IL-2 receptor or via direct binding to cytokines such as IL-4 and IL-15 (238–241).

Similar to T cells, also DC differentiation and maturation is inhibited by gangliosides through inhibition of NFκB signaling (242, 243). Besides, brain-derived gangliosides inhibit MHC-II antigen presentation by monocytes (244). GM2 and GM3 shed by melanomas were potent inhibitors of Fc receptor expression on monocytes and macrophages whereas GM1 and GD3 inhibited IL-1β production (245). Similarly, GM2 and GM3 were potent inhibitors of NK cell activity. Since GM2 showed reduced effector-target cell binding and GM3 did not, they are likely to act through different mechanisms (246). IL-3 dependent proliferation of BMMCs was inhibited by GM3, but in contrast to earlier proposed mechanisms, the authors excluded direct GM3-IL-3 interaction. However, it remains unknown whether the mechanism may be through competition with IL-3 for the IL-3 receptor (247). In summary, high concentrations of gangliosides shed by tumors lead to a downregulation of the cellular immune response.

Conversely, microglia downregulate TLR4 while upregulating TLR2 in the presence of free gangliosides, which thus contribute to inflammatory conditions in the brain (248). However, the mechanism by which gangliosides affect the microglial phenotype and whether this actually contributes to an inflammatory state in the brain has yet to be established.

## Targeting GSLs: Opportunities for Treatment

### Targeting of GSLs Using Antibodies/CAR T Cells

Since tumors often upregulate GSL expression, as discussed in the previous chapter, the 75 cancer antigen priorities of the National Cancer Institute at Rockville (USA) lists 4 different GSLs (249). The first one on the list is GD2, for which an antibody (dituximab beta; ch14.18/CHO) is currently being tested in phase III trials for patients with neuroblastoma (trial NCT01704716). Additionally, chimeric antigen receptors (CARs) have been designed and overexpressed in T cells to target GD2 overexpressing neuroblastoma (250–252). Next, an anti-GD3 antibody-drug conjugate (PF-06688992) is in a Phase I clinical trial for patients with stage III or IV melanoma (trial NCT03159117). Also for this GSL-target, CARs have been developed (253). Fucosyl-GM1 is being targeted by the antibody BMS-986012 that is currently tested in the preclinical phase with the goal to treat patients with small-cell lung carcinoma (254). The last GSLs on the list is GM3 for which an antibody is undergoing preclinical investigation by Morphotek.

Yet another option is to vaccinate with GSLs or structures that bear GSL antigens in order to induce an antibody response toward the GSLs overexpressed by a patient's tumor. The disadvantage, however, is that vaccinations with carbohydrates require (a lot of) purified carbohydrates and often result in CD4^+^ T cell independent low affinity IgM responses without long-lived B cell memory (255). To overcome these challenges, either purified carbohydrates or synthetic polymers harboring the epitope can be fused to carrier proteins (e.g., keyhole limpet hemocyanin or tetanus toxoid) that are able to induce CD4^+^ T cell activation. Since conjugation of carbohydrate epitopes to proteins is hard to control, fully synthetic vaccines are being developed (256).

### Inhibition of GSL Synthesis to Active Immune Cells

In 2003 and 2014 the UGCG inhibitors Miglustat [N-butyl 1-deoxynojirimycin (NBDNJ)] and Eliglustat, respectively, received FDA approval for treatment of Gaucher disease in order to prevent accumulation of GlcCer in these patients. Until 1994, NBDNJ was described to inhibit α-glucosidases in the *N*-glycosylation pathway. *In vitro* work on purified proteins shows that the IC_50_ for NBDNJ was 0.57 μM for α-glucosidase I and 20.4 μM for UGCG. However, due to localization of UGCG on the cytoplasmic side and α-glucosidase I on the luminal side of the ER, a 10-fold lower concentration NBDNJ is required to inhibit UGCG compared to α-glucosidase I in intact cells (257–259). For long it has been hypothesized that inhibitors of GSL synthesis like NBDNJ could also be beneficial for other diseases including cancer (260).

In several mouse models it has been shown that inhibition of GSL synthesis decreases tumor load or even cured the mice (261). Moreover, in a multiple myeloma mouse model, inhibition of GSL synthesis decreased osteoclast activation and thereby the osteolytic lesions that are often present in multiple myeloma patients (262). Since it is even suggested that aberrant GSL synthesis by tumors cause drug-resistance (263, 264), inhibiting GSL synthesis would be great for a combination therapy. Apart from drug-resistance, high expression of GSLs by tumors also negatively affects T cell and DC function, so GSL synthesis inhibition could also be beneficial for cancer immunotherapies.

However, in a Phase I trial where NBDNJ was administered to HIV patients it was found that some patients developed borderline or transient leuko- or neutropenia that was unrelated to dosage (265). In addition, GSL inhibitors may have a negative effect on lymphocyte development and maturation *in vivo* (266), In the case of anti-tumor treatment, however, the patient population would only have a temporary inhibition of GSL synthesis and a functional immune system. Additionally, studies in patients suffering from Gaucher disease do not mention any immune-related side-effect of NB-DNJ (267–269). In this review, we discussed several functions of the immune system that rely on GSLs, therefore it is likely that some functions may be impaired by GSL synthesis inhibitors and their off-label use should be well-substantiated.

## Concluding Remarks

It is clear by now that GSLs are important constituents of a functional immune system. GSLs play versatile roles in physiology and pathophysiology. The knowledge on these roles is largely skewed by the limitations of the tools available. Still, investigators have discovered on a molecular level that GSLs are essential for the recruitment of (immune-related) proteins to specific membrane microdomains and that GSLs can directly interact with surface receptors. Interactions directly with molecules on other cell types further shape the multi-faceted function of GSLs in immunity. We believe that these GSL functions are closely interconnected to control immune cell function through dynamic regulation of GSL composition. As a consequence, various pathologies are highly related to specific GSL repertoires. We therefore also provided a brief summary of the therapeutic opportunities of GSL synthesis dysregulation that are currently being evaluated. New mechanistic insights in the (immunological) functions of GSLs in health and disease will allow to expand the described options and applications. Available state-of-the-art technologies will be of great help to take the field a great leap forward. Specifically, a validated gRNA library to target all known human GTs by CRISPR/Cas9 has been recently constructed (270). Difficulties of introducing the CRISPR/Cas9 machinery into primary immune cells, such as B and T cells, have also been overcome by electroporation protocols and the usage of recombinant gRNA-loaded Cas9 (271, 272). Furthermore, the development and combination of high-sensitive analytical platforms based on mass spectrometry have boosted the detection of less common GSL-species. And the current throughput and analysis efficiency allows for comprehensive profiling, quantification, and structural characterization of GSLs extracted from tissues and cells (48, 273–275). All these advancements allow the community to systemically investigate the role of individual GSLs in immune cells.

## Author Contributions

TZ and AdW contributed equally to the writing. MW and RS conceived and edited the manuscript. All authors read and approved the final manuscript.

### Conflict of Interest Statement

The authors declare that the research was conducted in the absence of any commercial or financial relationships that could be construed as a potential conflict of interest.

## Abbreviations

GSL: glycosphingolipid
GT: glycosyltransferase
GEMs: glycosphingolipid enriched microdomains
Cer: ceramide
UGCG: UDP-glucose ceramide glycosyltransferase
B4GALT: β1,4-galactosyltransferase
GlcCer: glucosylceramide
LacCer: lactosylceramide
ST3GAL: α2,3-sialyltransferase
B4GALNT1: β1,4-*N*-acetylgalactosaminyltransferase 1
GalNAc: *N*-acetylgalactosamine
Lc3: lactotriaosylceramide
Gb3: globotriaosylceramide
isoGb3: isoglobotriaosylceramide
B3GNT: β1,3-*N*-acetylglucosaminyltransferase
A4GALT: α1,4-galactosyltransferase
A3GALT2: α1,3-galactosyltransferase 2
CD: cluster of differentiation
CTB: cholera toxin subunit B
BMMCs: bone marrow culture-derived mast cells
SMCs: serosal mast cells
PMA: phorbol myristate acetate
Le^x^, Lewis X structures: Galβ1-4(Fucα1-3)GlcNAcβ1-
S(3)nLc4: Neu5Acα2-3nLc4
S(6)nLc4: Neu5Acα2-6nLc4
S(3)nLc6: Neu5Acα2-3nLc6
moDCs: monocyte-derived dendritic cells
Galα1-3(F(2))ASGM1: Galα1-3(Fucα1-2)asialoGM1
Fo, Forssman glycolipid antigen: GalNAcα1-3Gb4
BMDCs: bone marrow-derived dendritic cells
NKT: natural killer T
NK: natural killer
NeuGc: *N*-glycolylneuraminic acid
LacNAc-GM1: Galβ1-4GlcNAcβ1-3GM1a
Galα1-3LacNAc-GM1, S(3)LacNAc-GM1: Neu5Acα2-3Galβ1-4GlcNAcβ1-3GM1a
IL: interleukin
IFN-α: Interferon-α
TNF-α: tumor necrosis factor-α
LPS: lipopolysaccharide
TCR: T cell receptor
LXR: liver X receptor
ST: shiga toxin
STb: B subunit of ST
PCI: protein-carbohydrate interaction
CCI: carbohydrate-carbohydrate interaction
EGFR: epidermal growth factor receptor
CAR: chimeric antigen receptor
TLR4-MD2: Toll-Like Receptor 4-myeloid differentiation factor 2
EtxB: enterotoxin subunit B
HIV: human immunodeficiency virus
STb: B subunit of ST
NBDNJ: *N*-butyl 1-deoxynojirimycin. Glycan abbreviations and structures are listed in Table S1.
