# Supplementary material for: The Role of Glycosphingolipids in Immune Cell Functions
Source: Front Immunol. 2019 Jan 29;10:90. doi: 10.3389/fimmu.2019.00090 (PMC6361815; doi:10.3389/fimmu.2019.00090)
Supplement: Supplementary file 1 [file Table_1.DOCX]

**Table S1. GSL abbreviations and their structures.**

| **Glucosylated and galactosylated ceramide precursors** | |
| --- | --- |
| GlcCer | Glcβ1-1Cer |
| LacCer | Galβ1-4Glcβ1-1Cer |
| GalCer | Galα1-1Cer |
| Galα1-2GalCer | Galα1-2Galα1-1Cer |
| **Gangliosides** | |
| GM3 | Neu5Acα2-3Galβ1-4Glcβ1-1Cer |
| GM2 | GalNAcβ1-4(Neu5Acα2-3)Galβ1-4Glcβ1-1Cer |
| GM1a | Galβ1-3GalNAcβ1-4(Neu5Acα2-3)Galβ1-4Glcβ1-1Cer |
| GD1a | Neu5Acα2-3Galβ1-3GalNAcβ1-4(Neu5Acα2-3)Galβ1-4Glcβ1-1Cer |
| GT1a | Neu5Acα2-8Neu5Acα2-3Galβ1-3GalNAcβ1-4(Neu5Acα2-3)Galβ1-4Glcβ1-1Cer |
| GD3 | Neu5Acα2-8Neu5Acα2-3Galβ1-4Glcβ1-1Cer |
| GD2 | GalNAcβ1-4(Neu5Acα2-8Neu5Acα2-3)Galβ1-4Glcβ1-1Cer |
| GD1b | Galβ1-3GalNAcβ1-4(Neu5Acα2-8Neu5Acα2-3)Galβ1-4Glcβ1-1Cer |
| GT1b | Neu5Acα2-3Galβ1-3GalNAcβ1-4(Neu5Acα2-8Neu5Acα2-3)Galβ1-4Glcβ1-1Cer |
| GQ1b | Neu5Acα2-8Neu5Acα2-3Galβ1-3GalNAcβ1-4(Neu5Acα2-8Neu5Acα2-3)Galβ1-4Glcβ1-1Cer |
| GT3 | Neu5Acα2-8Neu5Acα2-8Neu5Acα2-3Galβ1-4Glcβ1-1Cer |
| GT2 | GalNAcβ1-4(Neu5Acα2-8Neu5Acα2-8Neu5Acα2-3)Galβ1-4Glcβ1-1Cer |
| GT1c | Galβ1-3GalNAcβ1-4(Neu5Acα2-8Neu5Acα2-8Neu5Acα2-3)Galβ1-4Glcβ1-1Cer |
| GQ1c | Neu5Acα2-3Galβ1-3GalNAcβ1-4(Neu5Acα2-8Neu5Acα2-8Neu5Acα2-3)Galβ1-4Glcβ1-1Cer |
| GD1α | Neu5Acα2-3Galβ1-3(Neu5Acα2-6)GalNAcβ1-4Galβ1-4Glcβ1-1Cer |
| Asialo GM2 | GalNAcβ1-4Galβ1-4Glcβ1-1Cer |
| Asialo GM1 | Galβ1-3GalNAcβ1-4Galβ1-4Glcβ1-1Cer |
| Galα1-3(F(2))ASGM1 | Galα1-3(Fucα1-2)Galβ1-3GalNAcβ1-4Galβ1-4Glcβ1-1Cer |
| GM1b | Neu5Acα2-3Galβ1-3GalNAcβ1-4Galβ1-4Glcβ1-1Cer |
| GD1c | Neu5Acα2-8Neu5Acα2-3Galβ1-3GalNAcβ1-4Galβ1-4Glcβ1-1Cer |
| GalNAcGM1b | GalNAcβ1-4(Neu5Acα2-3)Galβ1-3GalNAcβ1-4Galβ1-4Glcβ1-1Cer |
| LacNAc-GM1 | Galβ1-3GalNAcβ1-4Galβ1-3GalNAcβ1-4(Neu5Acα2-3)Galβ1-4Glcβ1-1Cer |
| Galα1-3LacNAc-GM1 | Galα1-3Galβ1-3GalNAcβ1-4Galβ1-3GalNAcβ1-4(Neu5Acα2-3)Galβ1-4Glcβ1-1Cer |
| Galα1-3(LacNAc)_2_-GM1 | Galα1-3(Galβ1-3GalNAcβ1-4)_2_Galβ1-3GalNAcβ1-4(Neu5Acα2-3)Galβ1-4Glcβ1-1Cer |
| S(3)LacNAc-GM1 | Neu5Gcα2-3Galβ1-3GalNAcβ1-4(Neu5Acα2-3)Galβ1-3GalNAcβ1-4Galβ1-4Glcβ1-1Cer |
| (LacNAc)_n_-GM1b | (Galβ1-3GalNAcβ1-4)_n_(Neu5Acα2-3)Galβ1-3GalNAcβ1-4Galβ1-4Glcβ1-1Cer |
| **(iso)globosides** | |
| Gb3 | Galα1-4Galβ1-4Glcβ1-1Cer |
| isoGb3 | Galα1-3Galβ1-4Glcβ1-1Cer |
| Gb4 | GalNAcβ1-3Galα1-4Galβ1-4Glcβ1-1Cer |
| isoGb4 | GalNAcβ1-3Galα1-3Galβ1-4Glcβ1-1Cer |
| Gb5 | Galβ1-3GalNAcβ1-3Galα1-4Galβ1-4Glcβ1-1Cer |
| Fo | GalNAcα1-4GalNAcβ1-3Galα1-4Galβ1-4Glcβ1-1Cer |
| **(neo)lacto-series GSLs** | |
| Lc3 | GlcNAcβ1-3Galβ1-4Glcβ1-1Cer |
| Lc4 | Galβ1-3GlcNAcβ1-3Galβ1-4Glcβ1-1Cer |
| nLc4 | Galβ1-4GlcNAcβ1-3Galβ1-4Glcβ1-1Cer |
| S(3)nLc4 | Neu5Acα2-3Galβ1-4GlcNAcβ1-3Galβ1-4Glcβ1-1Cer |
| S(6)nLc4 | Neu5Acα2-6Galβ1-4GlcNAcβ1-3Galβ1-4Glcβ1-1Cer |
| S(3)nLc6 | Neu5Acα2-3Galβ1-4GlcNAcβ1-3Galβ1-4GlcNAcβ1-3Galβ1-4Glcβ1-1Cer |
| Desialylated dodecasaccharide ceramide | Galβ1-4GlcNAcβ1-3Galβ1-4(Fucα1-3)GlcNAcβ1-3Galβ1-4GlcNAcβ1-3Galβ1-4GlcNAcβ1-3Galβ1-4Glcβ1-1Cer |
| Dodecasaccharide ceramide | Neu5Acα2-3Galβ1-4GlcNAcβ1-3Galβ1-4(Fucα1-3)GlcNAcβ1-3Galβ1-4GlcNAcβ1-3Galβ1-4GlcNAcβ1-3Galβ1-4Glcβ1-1Cer |
